# Relative T2-FLAIR signal intensity surrounding residual cavity is associated with survival prognosis in patients with lower-grade gliomas

**DOI:** 10.3389/fonc.2022.960917

**Published:** 2022-09-15

**Authors:** Tao Yuan, Zhen Gao, Fei Wang, Jia-Liang Ren, Tianda Wang, Hongbo Zhong, Guodong Gao, Guanmin Quan

**Affiliations:** ^1^ Department of Medical Imaging, The Second Hospital of Hebei Medical University, Shijiazhuang, China; ^2^ Department of Pharmaceuticals Diagnostics, General Electric Healthcare China, Beijing, China; ^3^ Department of Radiology, People’s Hospital of Tangshan City, Tangshan, China

**Keywords:** lower-grade glioma, survival, prediction, magnetic resonance imaging, FLAIR = fluid-attenuated inversion recovery

## Abstract

**Aims:**

To investigate whether the relative signal intensity surrounding the residual cavity on T2-fluid-attenuated inversion recovery (rFLAIR) can improve the survival prediction of lower-grade glioma (LGG) patients.

**Methods:**

Clinical and pathological data and the follow-up MR imaging of 144 patients with LGG were analyzed. We calculated rFLAIR with Image J software. Logistic analysis was used to explore the significant impact factors on progression-free survival (PFS) and overall survival (OS). Several models were set up to predict the survival prognosis of LGG.

**Results:**

A higher rFLAIR [1.81 (0.83)] [median (IQR)] of non-enhancing regions surrounding the residual cavity was detected in the progressed group (n=77) than that [1.55 (0.33)] [median (IQR)] of the not-progressed group (n = 67) (P<0.001). Multivariate analysis showed that lower KPS (≤75), and higher rFLAIR (>1.622) were independent predictors for poor PFS (P<0.05), whereas lower KPS (≤75) and thick-linear and nodular enhancement were the independent predictors for poor OS (P<0.05). The cutoff rFLAIR value of 1.622 could be used to predict poor PFS (HR = 0.31, 95%CI 0.20–0.48) (P<0.001) and OS (HR = 0.27, 95%CI 0.14–0.51) (P=0.002). Both the areas under the ROC curve (AUCs) for predicting poor PFS (AUC, 0.771) and OS (AUC, 0.831) with a combined model that contained rFLAIR were higher than those of any other models.

**Conclusion:**

Higher rFALIR (>1.622) in non-enhancing regions surrounding the residual cavity can be used as a biomarker of the poor survival of LGG. rFLAIR is helpful to improve the survival prediction of posttreatment LGG patients.

## Introduction

Lower-grade gliomas (LGGs), including World Health Organization (WHO) grade II and III gliomas, are actually a heterogeneous group of cerebral primary neoplasm with highly variable overall survival (OS) varied from 2.7 to 16.7 years (1, 2). Since the relative lower incidence of the enhancement of LGG, the follow-up MRI assessment is primarily based on T2-weighted fluid-attenuated inversion recovery (FLAIR) findings, whereas the high signal on FLAIR may be due to various underlying pathological changes, the high signal in the non-enhancing region surrounding the residual cavity is still a challenge to interpret (3).

In the new version of Response Assessment in Neuro-Oncology (RANO), the RANO-LGG criteria was developed. FLAIR and contrast-enhanced T1WI (CE-T1WI) were still the basic sequences in the follow-up MRI protocol of posttreatment LGG. The response and progression of lesions were determined mainly by the decrease and increase of perpendicular diameters of FLAIR high-signal regions outside the residual cavity separately (4). Bette et al. assessed the overall FLAIR high-signal volume *via* manual segmentation (3). They found that early FLAIR volume evolution is an independent factor of LGG progression. Continuous follow-up MRIs, for example, every 3 months or at a longer interval, is a precise method for the discrimination of the residual tumor or progression from other pathologic changes. However, the strategy “wait and see” needs a long time to follow up and may lead to psychological torture to patients and the delay of salvage therapy. On the other hand, the measurement of the diameter or volume could not comprehensively reflect the pathophysiologic changes of the residual FLAIR high signal, because the high signal outside the residual cavity may not only be related to residual tumor but also to non-specific postoperative changes as well as ischemia. Previous studies suggested that functional MR techniques, such as perfusion imaging, proton MR spectroscopy (MRS), diffusion tensor imaging (DTI), and amide proton transfer (APT) imaging may be useful in distinguishing these conditions (5, 6), whereas the application of advanced MR sequences is limited by their vague results as well as not being a routine exam in the clinic practice.

Therefore, the inability of the traditional visual inspection and FLAIR evolution for detecting the early progression of LGG would lead to the delay of the potential survival-improving treatment strategies. We hypothesized that a quantitative method would be useful to characterize the evolution of residual FLAIR high signal. To the best of our knowledge, the role of the quantitative metrics of FLAIR high signal outside the residual cavity in diagnosing the survival outcome in LGG patients has not been investigated. In the present study, we retrospectively compared the relative signal intensity of FLAIR surrounding the residual cavity (rFLAIR) between the progressed and not-progressed groups of posttreatment LGG patients. Additionally, we evaluated the ability of rFLAIR in improving survival prediction in a combined prognosis model.

## Methods

### Study population and data collection

This study was approved by the ethical committee of The Second Hospital of Hebei University. For its retrospective nature, informed consent from patients was waived.

The study population included LGG patients who received treatment at our hospital for histologically confirmed astrocytoma and oligodendroglioma in grades II and III, and they were treated according to the guideline of the National Comprehensive Cancer Network (NCCN) (7) between April 2014 and December 2019. The pathological diagnosis of these LGG patients was made based on the 2007 WHO Classification of Tumors of the Central Nervous System. Astrocytoma was diagnosed by the presence of isocitrate dehydrogenase (IDH) mutation, p53, and alpha-thalassemia/mental retardation syndrome X-linked (ATRX) mutation. Oligodendroglioma was diagnosed when IDH mutation and short chromosome 1 and long chromosome 19 arms (1p19q) codeletion were detected (8).

The inclusion criteria of participants were: (1) gross total resection of tumors. Since there is often no or less prominent enhancement of the tumor after contrast medium injection, the extent of resection of LGG in the present study included both enhanced and FLAIR high-signal areas (9). (2) With age older than 18 years old (10) and (3) had undergone at least six times follow-up MR exams, including <72 h after operation, and before and after radiotherapy, at 3 months, 6 months, and >12 months after the completion of radiotherapy. (4) Followed up for more than 12 months after treatment. The exclusion criteria were: (1) only partial resection or biopsy was made; (2) with age less than 18 years old; (3) with follow-up <12 months; (4) with poor image quality; (5) without high signal surrounding the residual cavity; (6) had not received standardization treatment according to NCCN or had antiangiogenetic therapies after operation; and (7) the patient died from causes other than the radiological or clinical deterioration of LGG.

**Figure 1:**
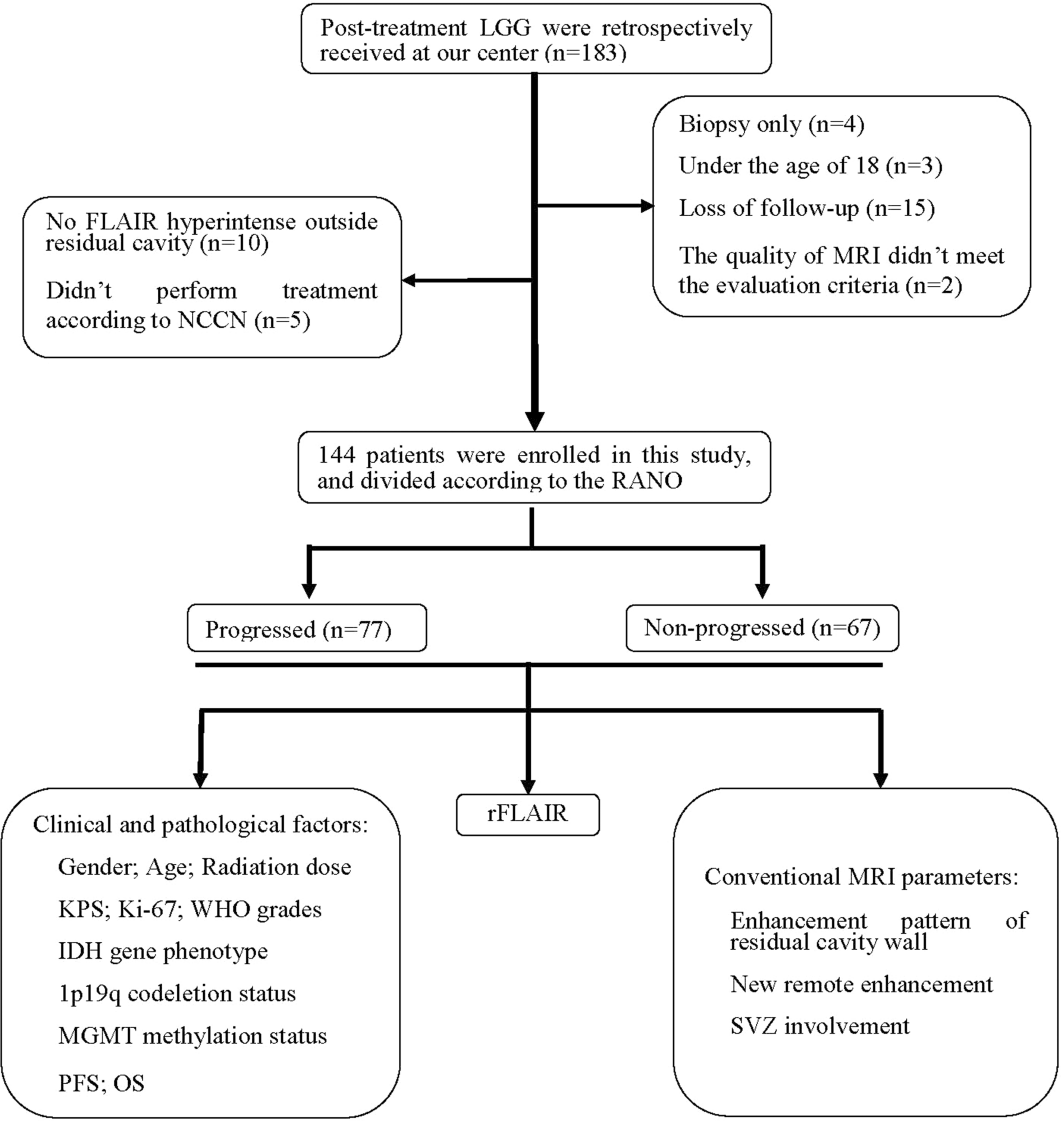
Patient flowchart. IDH, isocitrate dehydrogenase; KPS, Karnofsky Performance Scale; LGG, lower-grade glioma; MGMT, oxygen 6-methylguanine-DNA methyltransferase; NCCN, National Comprehensive Cancer Network; OS, overall survival; PFS, progression-free survival; RANO, Response Assessment in Neuro-Oncology; rFLAIR, relative FLAIR; SVZ, subventricular zone.

The patients’ data, including demographics, pathologic diagnosis, treatment schedule, MR imaging data, and clinical outcomes were collected from the hospital information system (HIS) (**Figure 1**). Clinical information obtained included the dates of tumor resection and chemoradiotherapy, the dates of progression, and postoperative Karnofsky Performance Scale (KPS). The collected pathologic information included the histological type and grade of tumors, antigen identified by the monoclonal antibody Ki-67, IDH mutation status, 1p19q status, and oxygen 6-methylguanine DNA methyltransferase (MGMT) promoter methylation status (11).

The LGG patients were divided into progressed within 18 months (abbreviated as “progressed”) and not progressed within 18 months (abbreviated as “not-progressed”) groups according to the RANO criteria (4). The disease progression for grade II gliomas was diagnosed when one of the following criteria was met: (1) the development of new lesions or increase of enhanced disease; (2) ≥25% increase in the sum of perpendicular diameters of the FLAIR abnormality; and (3) definite clinical deterioration. The definition of disease progression for grade III gliomas was based on one of the following criteria: (1) ≥25% increase in the sum of perpendicular diameters of the contrasted lesions; (2) increase of the FLAIR abnormality; (3) new enhancement disease; and (4) definite clinical deterioration. Those LGG patients who were dead ahead of the dead time of this study were ascribed to the progressed group (4). The time from the date of surgery to the date of the last follow-up or death was defined as OS. The time from the date of surgery to the date of progression or the date of the last follow-up without progression was defined as progression-free survival (PFS) (12).

### Magnetic resonance imaging acquisition and analysis

A 3-T MR scanner (PHILIPS MRI Systems, Achieva, Best, the Netherlands) was used for the serial follow-up MR imaging of all LGG patients. The follow-up MRI protocol included pre- and postcontrast transverse T1-weighted imaging (T1WI), transverse and sagittal T2-weighted imaging (T2WI), FLAIR, and postcontrast axial and sagittal and coronal T1WI. The postcontrast T1WI (CE-T1WI) was made after the injection of a standard dose (0.1 mmol per kilogram of body weight) of gadobutrol (Gadovist, Bayer Schering Pharma, Berlin, Germany) at a rate of 3 ml/s. A follow-up MR examination was performed within 72 h after tumor resection, before and at the end of radiotherapy, and 3 and 6 months after radiotherapy. Thereafter, follow-up MR was made every 1~3 months depending on the enhanced disease and the increase of high-signal lesions on FLAIR images.

All MRI imaging data were collected from the picture archiving and communication system. The conventional MR features analyzed included: the enhancement pattern of the residual cavity wall, new distal enhancement disease, and new involvement of the subventricular zone (SVZ). The enhancement of the residual cavity wall was categorized into three types: no enhancement; thin-linear enhancement (partial or entire wall enhancement with thickness <3 mm), thick-linear (partial or entire wall enhancement of 3~5 mm in thickness) or nodular (5~10 mm in thickness) enhancement (13). New distal parenchymal enhancement was defined as a newly enhanced disease that is not contiguous (>1.5 cm away) with a residual cavity or remnants of tumor after resection (14). New SVZ involvement was defined as new enhancement lesions after standardized treatment on follow-up MRI.

**Figure 2:**
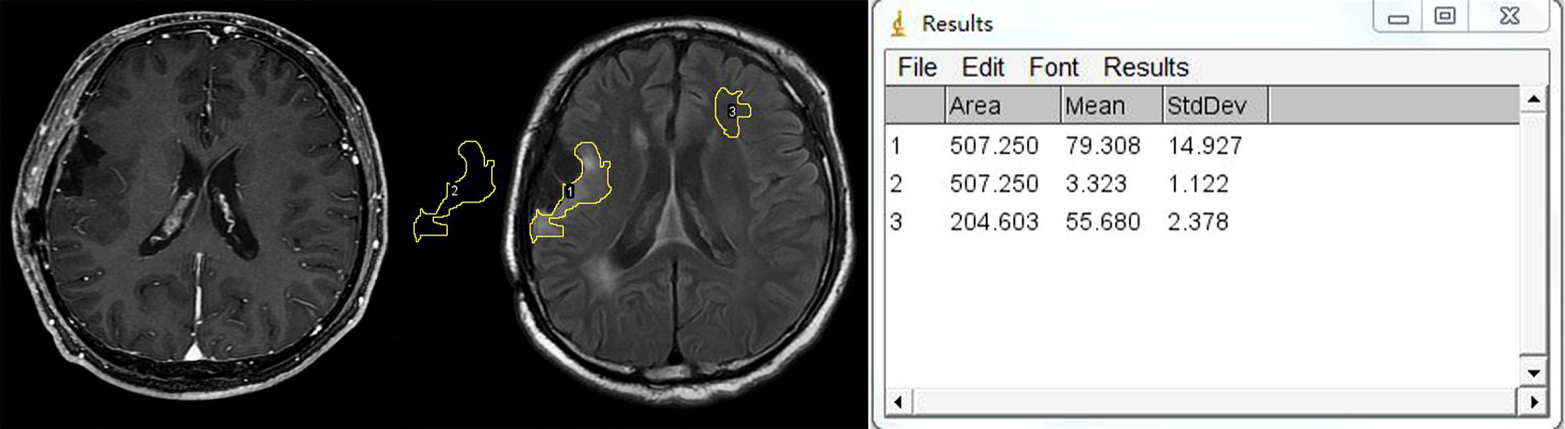
Example of region of interest (ROI) placement. Left figure: CE-T1WI shows no enhancement surrounding the residual cavity. Middle figure shows that three ROIs are placed in the hyperintensity lesion surrounding the residual cavity, contralateral white matter, and background of the image separately. Right figure shows the measurement output of Image J.

The relative FLAIR signal intensity was measured on the axial section with the largest area of high signal at the first follow-up MR exam after the completion of radiotherapy. All measurements were made with an open-access image software, Image J (http://rsbweb.nih.gov/ij/docs/guide/). After the input of FLAIR images into the software, we measured the signal intensity in the following three regions of interest (ROIs): all high-signal regions surrounding the residual cavity, contralateral cerebral white matter without abnormal signal intensity, and the background of the image (**Figure 2**). The average value of the signal intensity was recorded. rFLAIR was calculated as the following formula: rFLAIR = (the gray intensity of the high-signal region surrounding the residual cavity – the gray intensity of the background)/(the gray intensity of the contralateral cerebral white matter without abnormality – the gray intensity of the background of the image) (15). The imaging findings and analyses of follow-up MRI were made independently by two neuroradiologists (with 6 and 18 years of experience in diagnostic radiology). When a disagreement existed, consent was reached after consulting another neuroradiologist with 26 years of experience in neuroradiology.

### Statistical analysis

Statistical calculations were performed with IBM SPSS Version 21 software and GraphPad Prism 8. Quantitative variables that were consistent with the normal distribution were reported as mean ± standard deviation. Variables with non-normal distributions were reported as median and interquartile range. The normal and non-normal distribution of variables was differentiated with the Kolmogorov–Smirnov test using SPSS version 21.0 software. The variables were considered as normal distribution if *P* > 0.05. Otherwise, the variables were in non-normal distribution. PFS and OS were created using the Kaplan–Meier method and reported as 95% confidence interval (CI). Categorical variables were compared with the chi-square test between the progressed and not-progressed groups, including quantitative clinical factors and conventional MRI findings. For comparing the difference of quantitative variables with normal distribution between progressed and not-progressed groups, we employed a two-independent-sample *t*-test. Otherwise, the Mann–Whitney *U* test was used for the comparison of non-normal distribution variables. The difference in the survival curve between different groups was compared using the results of the log-rank test. Univariate and multivariate Cox regression analyses were made for evaluating the risk factors for poorer survival and reported as a hazard ratio (HR) in the form of 95% CI. Those characters with *P* < 0.05 in the comparison between progressed and not-progressed groups were selected for univariate regression analysis. Then, we selected the variables with *P* < 0.05 in univariate analysis either for PFS or OS in multivariate regression analysis. Receiver operating characteristic (ROC) curve analysis was employed to determine the threshold value and evaluate the diagnostic performance of different prognosis models with the area under the curve (AUC), accuracy, sensitivity, and specificity. The cutoff value of age, radiation dose, KPS, Ki-67, and rFLAIR for discriminating progressed and not-progressed patients was determined when Youden’s index was highest. *P*-values less than 0.05 were considered statistically significant. Interreader variability in MR images was analyzed with the intraclass correlation coefficient (ICC).

The agreement was excellent between the two neuroradiologists for the evaluation of MRI findings, including the enhancement pattern of the residual cavity wall (ICC, 0.976; 95%CI 0.967–0.983), new distal enhancement (ICC, 0.938; 95%CI 0.914–0.955, P<0.001), and new SVZ involvement (ICC, 0.959; 95%CI 0.943–0.971, P<0.001).

## Results

### Patient characteristic

In the 183 LGG patients initially screened, 39 patients were excluded for the following reasons: diagnosed with biopsy (*n*=4), age less than 18 years old (*n*=3), with incomplete MRI follow-up data (*n*=15), with poor image quality (*n*=2), without high signal outside residual cavity (*n*=10), and had not received standardization treatment according to NCCN (*n*=5). Finally, 144 patients were enrolled in this study, including 62 women and 82 men, aged from 18 to 73 years old (44.87 years ±12.46). Among the 144 patients, there were 54 patients with grade II gliomas (25 with oligodendroglioma, 29 with astrocytoma), and 90 patients with grade III gliomas (39 with anaplastic oligodendroglioma, 51 with anaplastic astrocytoma). The gene phenotypes of these 144 patients were: isocitrate dehydrogenase (IDH) mutation status (92 patients, including 47 with IDH mutation and 45 with wild type), short chromosome 1 and long chromosome 19 arms (1p19q) status (92 patients, including 40 with codeleted, and 52 without codeleted), oxygen 6-methylguanine-DNAmethyltransferase (MGMT) and promoter methylation status (46 patients, including 33 with positive methylation and 13 without methylation). The enhancement patterns of the residual cavity wall included: 94 patients without enhancement or with thin linear enhancement and 50 patients with thick linear or nodular enhancement. New distal parenchymal enhancement was found for 8 patients and new SVZ involvement for 30 patients. There was no contrast enhancement lesion found in the high signal region outside the residual cavity on FLAIR imaging in 115 patients (79.86%) in the first follow-up MRI after radiotherapy. The median follow-up time was 806 days (95% CI 584–1,089 days). The median PFS was 786 days (95% CI 594–978 days), and the median OS was 1,608 days (95% CI 1,443–1,774 days). 40 patients (27.78%) were dead during the follow-up period.

### Comparison between progressed and not-progressed groups

The following variables in the progressed group were higher than those in the not-progressed group: Ki-67 (0.15 vs. 0.10, *P*=0.005), grade III tumors 61.1% vs. 38.9%, *P*=0.018), wild-type IDH phenotype (73.3% vs. 26.7%, *P*<0.001), thick-linear or nodular enhancement of residual cavity wall (74.0% vs. 26.0%, *P*<0.001), new remote enhancement (100.0% vs. 0.0%, *P*=0.007), new SVZ involvement (83.3% vs. 16.7%, *P*<0.001), and rFLAIR (1.81 vs. 1.55, *P*<0.001). The KPS score (90 ± 4) and the incidence of the 1p19q co-deletion of the not-progressed group (75.0%) were higher than those of the progressed group (87 ± 7 and 42.3% separately) (*P*=0.016, *P*=0.002). The PFS of the progressed group (median, 431 days) was shorter than that of the not-progressed group (median, 831 days) (*P*<0.001), whereas there was no significant difference on the OS between the progressed and not-progressed groups (791 days vs. 831 days, *P*=0.327), neither for gender (*P*=0.959), age (*P*=0.053), radiation dose ((*P*=0.095), MGMT methylation status (*P*=0.425), and the increase of the perpendicular diameters of FLAIR lesions (*P*=0.156) between the two groups (**Table 1**).

**Table 1:** Baseline characteristic of posttreatment patients with lower-grade glioma (LGG; n=144).

Characteristic	Not-progressed (n=67)	Progressed (n=77)	p-value
**Sex (%),** Male	38 (46.3)	44 (53.7)	0.959
**Age (years old)**	42.74 ± 11.75	46.74 ± 12.82	0.053
**Radiation dose**	59.92 (6.00)	60.00 (0.08)	0.095
**KPS**	90 ± 4	87 ± 7	0.016
**Ki-67 (%)**	0.10 (0.20)	0.15 (0.20)	0.005
**WHO grades (%)**			
WHO grade II	32 (59.3)	22 (40.7)	0.018
WHO grade III	35 (38.9)	55 (61.1)	
**Integrated diagnosis**			
Astrocytoma-IDH mut- WHO grade II	9 (69.2)	4 (30.8)	0.319 0.039
Astrocytoma-IDH wild- WHO grade III	4 (22.2)	14 (77.8)	
Oligodendroglioma-IDH mut- 1p19qcodel- WHO grade II	8 (80.0)	2 (20.0)	0.593
Oligodendroglioma-IDH mut- 1p19qcodel- WHO grade III	11 (91.7)	1 (8.3)	0.004
**New remote enhancements (%)**	0 (0.0)	8 (100.0)	0.007
**SVZ involvement (%)**	5 (16.7)	25 (83.3)	<0.001
**Increase FLAIR PD (%)**	13 ± 27	15 (32)	0.156
**rFLAIR**	1.55 (0.33)	1.81 (0.83)	<0.001
**PFS (days)**	831 (451)	431 (348)	<0.001
**OS (days)**	831 (451)	791 (605)	0.327

LGG, lower-grade glioma; Progressed, progressed within 18 months; Not-progressed, not progressed within 18 months; Gy, gray; IQR, interquartile range; KPS, Karnofsky Performance Scale; IDH, isocitrate dehydrogenase; MGMT, oxygen 6-methylguanine-DNA methyltransferase; SVZ, subventricular zone; rFLAIR, relative FLAIR; PFS, progression-free survival; OS, overall survival; Increase FLAIR PD, increase of the perpendicular diameters on FLAIR lesions.

### Comparison between oligodendroglioma and astrocytoma

Since the outcome of oligodendroglioma was considered more favorable than that of astrocytoma with the same WHO grade (16), we compared the survival outcome of these two tumors. In the present study, the PFS of patients with oligodendroglioma in grade II (1,210 days) was longer than that of patients with astrocytoma in grade II (843 days); the OS of oligodendroglioma patients in grade II (1,443 days) was shorter than that of astrocytoma patients in grade II (1,505 days). However, there was no significant difference between them (*P*=0.052 for PFS and *P*=0.750 for OS). Similarly, the PFS and OS of oligodendroglioma patients in grade III (786 and 1,516 days) were not different from those of astrocytoma patients in grade III (PFS, 564 days; OS, 1,077 days) (*P*=0.208 and *P*=0.286, separately) despite longer PFS and OS for oliodendroglioma patients (**Table 2**).

**Table 2:** The survival difference of oligodendroglioma and astrocytoma in LGG.

Histologic types/Grades	II		III	
	PFS	OS	PFS	OS
**Oligodendroglioma**	1,210	1,443	786	1,516
**Astrocytoma**	873	1,505	564	1,077
**p-value**	0.052	0.750	0.208	0.286

LGG, lower-grade glioma; PFS, progression-free survival; OS, overall survival.

### Prognostic utility of relative FLAIR and various models

**Figure 3:**
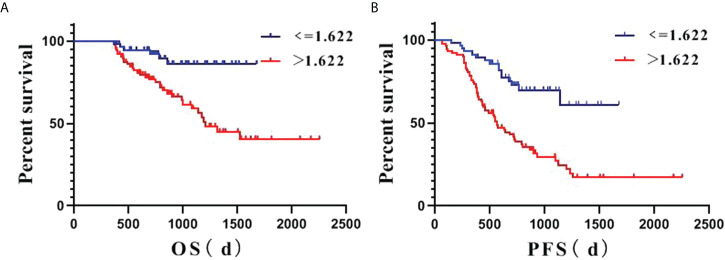
Kaplan–Meier estimate survival of posttreatment lower-grade glioma (LGG) patients based on the relative FLAIR (rFLAIR) cutoff value of 1.622. **(A)**, PFS. **(B)**, OS. d: day; LGG: lower-grade glioma; OS: overall survival; PFS: progression-free survival; rFLAIR: relative FLAIR.

Survival analysis based on rFLAIR (cutoff, 1.622) is shown in **Figures 3A, B**. Multivariate analysis showed that lower KPS (≤75), and higher rFLAIR (>1.622) were independent predictors for poor PFS (*P*<0.05), whereas lower KPS (≤75) and thick-linear and nodular enhancement were independent predictors for poor OS (*P*<0.05) (**Table 3**).

**Table 3:** Survival analysis of patients with LGG: univariate and multivariate analyses.

Characteristic	Univariate Analysis				Multivariate Analysis			
	PFS [HR(95%CI)] p-value		OS [HR(95%CI)] p*-*value		PFS [HR(95%CI)] p-value		OS [HR(95%CI)] p-value	
**KPS**	4.19[0.72;24.40]	0.007	2.68 [0.43;16.85]	0.086	0.95[0.92;0.99]	0.005	0.95[0.91;0.99]	0.010
**Ki-67**	0.68[0.42;1.08]	0.122	0.27[0.14;0.52]	0.003	0.48[0.09;2.62]	0.399	5.84[1.03;33.30]	0.047
**WHO grades**	0.52[0.33;0.81]	0.007	0.21[0.11;0.39]	<0.001	0.56[0.30;1.04]	0.065	0.44[0.15;1.30]	0.136
**Enhancement pattern of Residual cavity wall**	0.34[0.20;0.58]	<0.001	0.19[0.10;0.39]	<0.001	0.67[0.37;1.22]	0.190	0.37[0.17;0.82]	0.014
**New remote enhancement**	0.20[0.05;0.90]	<0.001	0.29[0.06;1.42]	0.006	0.59[0.25;1.40]	0.230	1.14[0.40;3.32]	0.804
**SVZ involvement**	0.32[0.17;0.62]	<0.001	0.23[0.10;0.56]	<0.001	0.66[0.35;1.24]	0.199	0.62[0.29;1.31]	0.207
**rFLAIR**	0.31[0.20;0.48]	<0.001	0.27[0.14;0.51]	0.002	1.64[1.12;2.42]	0.012	1.25[0.74;2.14]	0.41

LGG, lower-grade glioma; PFS, progression-free survival; OS, overall survival; HR, hazard ratio; CI, confidence interval; Gy, gray; KPS, Karnofsky Performance Scale; SVZ, subventricular zone; rFLAIR, relative FLAIR.

**Figure 4:**
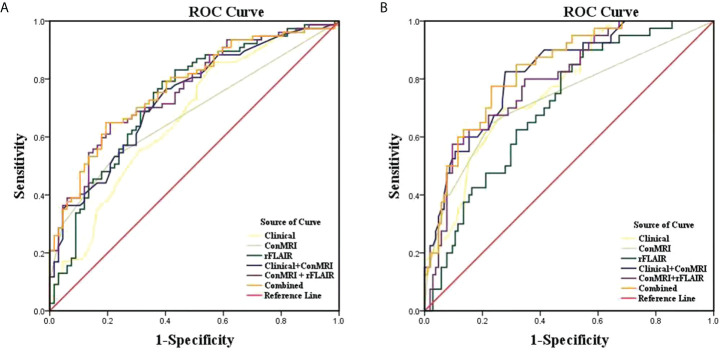
Receiver operating characteristic (ROC) analysis of different factors in the survival assessment of LGG patients. **(A)** ROC for PFS. **(B)** ROC for OS. Clinical: including age, radiation dose, KPS, Ki-67, and WHO grades; Combined: including clinical, Con MRI findings, and rFLAIR; Con MRI: including new remote enhancement, the enhancement pattern of the residual cavity wall, and SVZ; LGG: lower-grade glioma; OS: overall survival; PFS: progression-free survival; rFLAIR: relative FLAIR; ROC: receiver operating characteristic curve.

**Figure 5:**
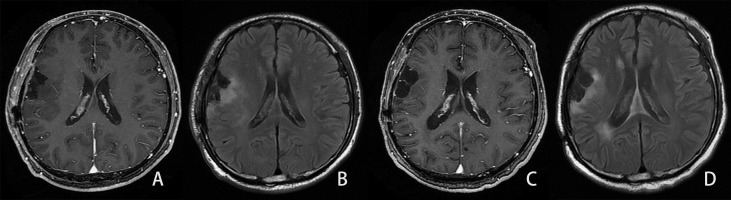
Example of the LGG patient in the not-progressed group. Same patient as shown in **Figure 2**. A man, 54 years old, with diffuse astrocytoma in the right frontal and parietal lobes, and an IDH mutant. The PFS and OS were 1,042 and 1,042 days, respectively. **(A, B)**. First follow-up MRI after the completion of radiotherapy. **(A)** Axial CE-T1WI does not show enhancement in the region corresponding to FLAIR hyperintensity. **(B)** Axial FLAIR images show the residual cavity and the surrounding hyperintensity lesion. C, **(D)** Follow-up MRI 28 months after the completion of radiotherapy. **(C)** CE-T1WI shows no obvious enhancement lesion. **(D)** Axial FLAIR images show similar hyperintensity surrounding the residual cavity compared to Figure B.

**Figure 6:**
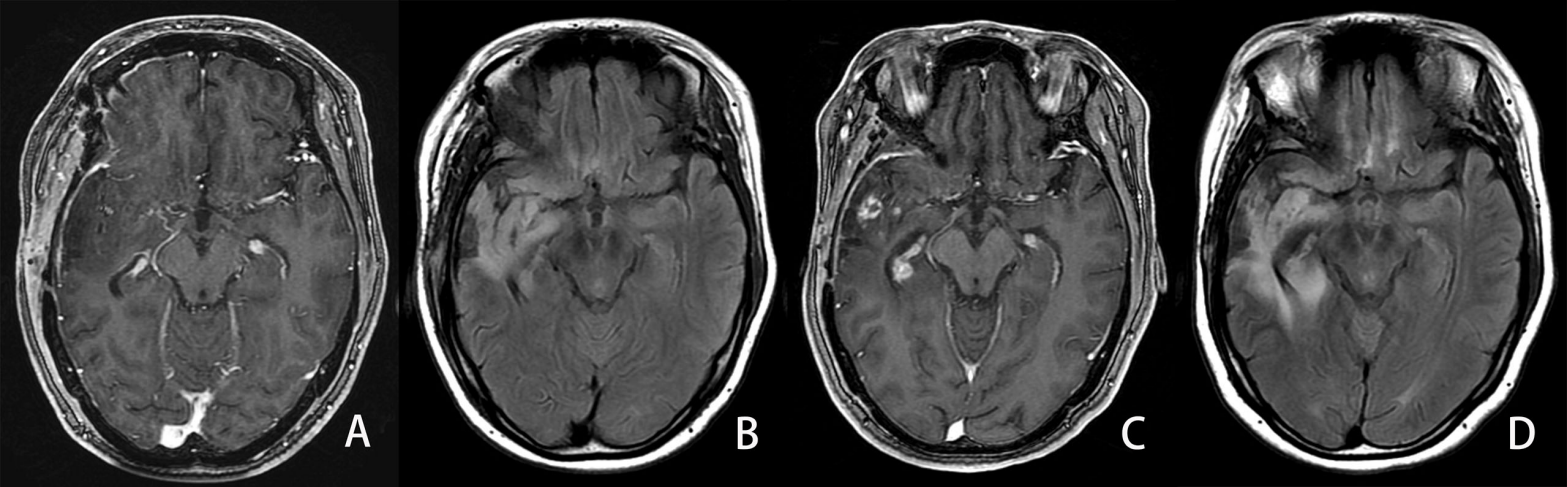
Example of an LGG patient in the progressed group. A 34-year-old man with diffuse IDH wild-type astrocytoma in his right temporal lobe. The PFS and OS were 339 and 1,035 days, respectively. A, **(B)** First follow-up MRI after the completion of radiotherapy. **(A)** Axial CE-T1WI does not show enhancement in the region corresponding to FLAIR hyperintensity. **(B)** Axial FLAIR images show the residual cavity and the surrounding hyperintensity lesion. C, **(D)** Follow-up MRI 25 months after the completion of radiotherapy. **(C)** CE-T1WI shows new developed ring and curved linear enhancement. **(D)** Axial FLAIR images show the prominent enlargement (42%) of the hyperintensity lesion surrounding the residual cavity.

ROC analysis showed the areas under the ROC curve (AUCs) for predicting poor PFS: clinical model, 0.667;conventional MRI model, 0.678; rFLAIR model, 0.735; clinical + conventional MRI model, 0.733; conventional MRI + rFLAIR model, 0.763; clinical + conventional MRI + rFLAIR combined model, 0.771. Similarly, the AUCs for predicting poor OS are the following: clinical model, 0.769; conventional MRI model, 0.734; rFLAIR model 0.706; clinical + conventional MRI model, 0.821; conventional MRI + rFLAIR, 0.793; clinical + conventional MRI + rFLAIR combined model, 0.831 (**Table 4**, **Figures 4A, B** **)**. These results demonstrated that the diagnostic performance of survival outcome prediction would be improved when adding rFLAIR to the combined model. **Figures 5A–D** and **Figures 6A–D** show the classic examples of LGG patients with non-enhancing high-signal lesions in the progressed and not-progressed groups.

**Table 4:** ROC curve analysis of different models in survival assessment of LGG patients.

Characteristic		PFS			OS	
	AUC	95%CI	p-value	AUC	95%CI	p-value
**Clinical**	0.667	0.578–0.756	0.001	0.769	0.688–0.849	<0.001
**Con MRI**	0.678	0.591–0.765	<0.001	0.734	0.635–0.832	<0.001
**rFLAIR**	0.735	0.653–0.818	<0.001	0.706	0.618–0.794	<0.001
**Clinical+Con MRI**	0.733	0.652–0.814	<0.001	0.821	0.748–0.894	<0.001
**Con MRI+rFLAIR**	0.763	0.687–0.840	<0.001	0.793	0.715–0.872	<0.001
**Combined**	0.771	0.695–0.847	<0.001	0.831	0.762–0.901	<0.001

LGG, lower-grade glioma; PFS, progression-free survival; OS, overall survival; AUC, area under the curve; CI, confidence interval; Clinical, including KPS, Ki-67, and WHO grades; Con MRI, new remote enhancement, the enhancement pattern of the residual cavity wall and SVZ involvement; rFLAIR, relative FLAIR; Combined, combination of clinical, Con MRI, and rFLAIR factors.

## Discussion

In this study, we analyzed the gray intensity outside the residual cavity on FLAIR images in LGG patients with gross total tumor resection. This primary study proved that it was feasible to use the open-source software Image J to measure the relative gray value of the FLAIR sequence in postoperative LGG patients. We showed that a higher rFLAIR value (>1.622) is an adverse prognostic factor for posttreatment progression and survival prognosis in LGG patients. Our data strongly supported the hypothesis regarding the ability of the semiquantitative metric rFLAIR in improving the survival prediction in a combined prognosis model. Thus, aside from the perpendicular diameters, the relative gray intensity outside the residual cavity on FLAIR images could be used in the evaluation of posttreatment LGG patients.

The present study showed that the open-source software Image J could offer semiquantitative metrics from FLAIR images with satisfactory reproducibility. As a widely used image processing platform, Image J has already been employed in biological image analysis for depicting weak signal variation beyond the naked eye (17). Image J had previously been used in the evaluation of pontine glioma. In a series of 121 pediatric patients with posttreatment diffuse intrinsic pontine glioma (DIPG), Poussaint et al. generated quantitative metrics based on FLAIR images and ADC maps (18). They demonstrated that preradiotherapy FLAIR skewness and standard deviation were associated with shorter PFS. In the new version of the WHO classification of CNS tumors (5th edition, 2021) (18, 19), DIPG would mainly be a diffuse midline glioma with H3K27-altered, which is a kind of high-grade glioma with poor prognosis. In comparison, we analyzed the rFLAIR of LGG in cerebral parenchyma in the present study. However, both our results and the study of Poussaint et al. demonstrated the usefulness of Image J in the quantitative analysis of FLAIR images in posttreatment gliomas with different grades. There are several studies with similar technologies focusing on high-grade glioma or GBM, including one conducted by our team. In a study of 26 postoperative GBM patients (20), Chang et al. found that the areas of future GBM recurrence exhibit significant differences in signal intensity on FLAIR images months before the development of abnormal enhancement occurs. Since the incidence of enhancement in postoperative LGG patients is relatively lower, the identification of progression and other pathological changes in the high-intensity region of FLAIR has a greater value in predicting progression and prognosis. In the previous study of our team, we evaluated the prognostic value of the FLAIR signal intensity of the postoperative cavity on the survival state of high-grade glioma (HGG). We measured the signal intensity of the residual cavity rather than the hyperintensity area outside the cavity (15). Compared to these studies of high-grade glioma (HGG) or GBM patients, in the present study, we focused on the prediction of the progression and outcome of postoperative LGG patients.

Our method has several advantages. Firstly, we calculated the relative signal gray value by comparing the gray intensity of high-signal lesions outside the residual cavity with the contralateral white matter as well as background of the image, instead of directly measuring the signal intensity on certain MR equipment. Secondly, tumor progression after LGG treatment was evaluated according to the RANO criteria, which were mainly based on FLAIR disease. LGG, especially grade II gliomas, often manifested as FLAIR high-signal lesions without enhancement after gadolinium contrast medium injection (4). Therefore, the evaluation of these non-enhancing FLAIR high-signal lesions is critical for the therapy regimens of LGGs (3). Thirdly, there was a significant difference of rFLAIR between the progressed (1.81) and not-progressed (1.55) groups in this study. The rFLAIR and the combined model including rFLAIR could effectively predict poor survival outcomes. Therefore, rFLAIR may be an adequate surrogate metric when a suspected non-enhancing FLAIR high-signal lesion is found. Finally, antiangiogenic agents, including bevacizumab and cediranib, have been used in the treatment of gliomas. These agents may lead to a pseudoresponse temporally decreasing the permeability of the blood–brain barrier and consequently diminishing contrast enhancement (21). Therefore, FLAIR imaging could be more suitable to identify early tumor progression for detecting the increase of non-enhancing high- signal lesions and evert the influence of a pseudoresponse.

Higher rFLAIR surrounding the residual cavity is probably due to neoplastic cell infiltration or tumor remnant except edema. In one study that included 10 patients with WHO grade II–IV gliomas, Amjad et al. investigated the peri-tumoral high-signal regions on FLAIR imaging with functional MR techniques and targeted biopsy on 10 patients with WHO grade II–IV gliomas (22). They found tumor cell infiltration and a tumor core in 75% samples in FLAIR high-signal regions. Thus, tumor cells tend to infiltrate and manifest as non-enhancing FLAIR high-signal lesions. Although LGG is less aggressive than glioblastoma, Amjad et al. still confirmed that a portion of the tumor extended outside the gadolinium contrast-enhancing border in seven patients with WHO grade II and III gliomas. These non-contrast-enhancing lesions could be visualized well on FLAIR imaging (23). On the other hand, Chang et al. investigated the signal intensity outside the residual cavity on FLAIR imaging and found that small but significant changes could be detected months before the development of abnormal contrast-enhancing lesions (20). We also confirmed that new enhancing lesions developed on follow-up MRI within the earlier non-contrast-enhancing high-signal region on FLAIR imaging in 29 patients with LGG (20.1%) in the present study. For standardizing the intensity value of FLAIR images among patients, Chang et al. employed a histogram normalization algorithm (20), whereas we normalized the measurement of signal intensity on FLAIR images by comparing the gray value of non-contrast-enhancing lesions with the contralateral white matter as well as the background. The calculation method of rFLAIR in this study may be helpful to eliminate the influence of different scanning parameters and different magnetic fields on FLAIR imaging. rFLAIR in non-contrast-enhancing lesions outside the residual cavity of LGG can probably be used as an imaging marker for estimating the burden of microscopic non-enhancing tumors and predicting the location of recurrent disease in posttreatment LGG patients.

Moreover, we confirmed the prognostic prediction of other previously described MRI features (4, 9, 24), including the enhancement types of the residual wall, new distal enhancement, and new SVZ involvement. However, the prediction performance of these features was relatively lower (AUC of 0.678 for PFS, AUC of 0.734 for OS) and would be improved when combined with rFLAIR (AUC of 0.763 for PFS, AUC of 0.793 for OS). This phenomenon may be explained by the gliomas enrolled with a lower grade in this study. The above-mentioned MRI features could be detected more often in those posttreatment glioblastoma patients (13, 14). The incidences of thick-linear and nodular enhancement (34.87%), new distal enhancement (5.26%), and new SVZ involvement (20.39%) were lower than those of glioma (51.52%, 25.43%, and 49.14% separately) (13, 14). On the contrary, LGG often manifested as ill-defined high-signal FLAIR lesions and without postcontrast enhancement because of the less invasiveness, less angiogenesis, and minimal disruption of the blood–brain barrier (4). The new involvement of SVZ could be manifested as both new enhancement and non-enhancing FLAIR high-signal lesions in the SVZ region. In this study, new SVZ involvement was detected as non-enhancing FLAIR high-signal lesions in 32.47% patients in the progression group (25/77). SVZ could increase invasiveness and the migratory potential because it is the source of tumor precursor stem cells (12, 24). Therefore, in the present study, the high incidence rate of SVZ in the progression group had an adverse effect on the survival outcomes of LGG patients.

Our result also confirmed that previously reported clinical factors, including age, postoperative KPS score, Ki-67 score, and tumor grade, were associated with the survival outcome of LGG (9, 11, 25, 26). In an analysis of 113 grade III glioma patients, Hong et al. found that the cutoff values of 51- and 55-year-olds were the prognostic impactors for PFS and OS separately (9). In the present study, we confirmed that the patients in the progressed group (46.74 years old) were older than those in the not-progressed group (42.74 years old). The prognostic value of the Ki-67 index for LGG was similar to that for glioblastoma since a higher Ki-67 index in LGG was associated with malignant transformation and a poor survival outcome (26). We also found that the Ki-67 index in the progressed group (0.15) was higher than that of the not-progressed group (0.10). However, our study revealed the superiority of the outcome diagnostic performance with the combination of conventional MRI and rFLAIR (AUC of 0.763 for PFS, and AUC of 0.793 for OS) to those of clinical factors (AUC of 0.667 for PFS, and AUC of 0.769 for OS). We further combined conventional imaging findings with the quantitative metric of FLAIR images, rFLAIR, which can be more reliable in differentiating progressed from not-progressed patients with non-enhancing high-signal lesions outside the residual cavity. This combination improved the prognostic prediction performance effectively. Therefore, we recommend that LGG patients with suspicious non-enhancing high-signal lesions on FLAIR images should additionally calculate semiquantitative metrics from FLAIR images.

Several limitations should be mentioned in the present study. First, the sample in this retrospective study was relatively small. We enrolled consecutive LGG patients who had operated in a period of 5 years. However, since the relative lower incidence (43.2% of all gliomas) and a comparative, more benign course, LGG was less often encountered and treated aggressively in clinical practice (27). Thus, the results of this study warrant further validation with a larger multicenter investigation. Validating the technique on different scanners and FLAIR protocols would also be needed in the future. Second, as a retrospective analysis, there may be a selection bias of the patients. A few cases were excluded because of lost follow-up, without the high signal of FLAIR images, without standard treatment and follow-up measurement, and so on. Third, the discrimination of progression from non-progression lesions was based on follow-up data except nine patients who were confirmed as having progression disease by reoperation. Fourth, there was a certain sampling bias. We measured high-signal lesions without discrimination tumor remnants from posttreatment cerebral edema, ischemic change. We confirmed that some FLAIR high-signal lesions were due to cerebral edema and ischemia based on the decrease in the size of the lesions with a long-term follow-up, whereas, as a consequence of the operation, ischemia plays an important role in inducing high signals on FLAIR imaging and probably leads to the overestimation of tumor remnants (28). On the other hand, rFLAIR was measured on a single slice with the highest FLAIR high-intensity region, which may lead to a potential sampling bias. Volumetric evaluation could be included as a future improvement of the technique. Similarly, it would be of interest to see if the longitudinal changes of rFLAIR could provide an additional prognostic value of LGG. Fifth, although we collected molecular pathological data from some patients, including the mutation of IDH, MGMT, and 1p19q co-deletion, a molecular pathology examination has not been widely included in routine clinical examination in the authors’, especially in the era before 2016. Recently, the Consortium to Inform Molecular and Practical Approaches to CNS Tumor Taxonomy working committee considered that histologic grade II and III IDH wild-type astrocytic gliomas should be referred to as diffuse astrocytic glioma, IDH-wild-type, for these gliomas contain high-level EGFR amplification or TERT promoter mutations (27, 29). Further analysis on the outcome evaluation of LGG in the light of FLAIR high- signal lesions should be based on the genetics of LGG in the future. Finally, since FLAIR imaging data should be downloaded and transferred to another computer for measuring the gray value and calculating rFLAIR; this method is not convenient in clinical practice. Therefore, we suggested that rFLAIR should be used as a subsidiary variable when progression and non-progression lesions in LGG patients could not be differentiated only based on conventional structural MRI findings.

## Conclusion

In conclusion, we found that the higher rFALIR (>1.622) of non-contrast-enhancing lesions surrounding the residual cavity was a useful predictor of the poor survival of LGG. As one reproducible, accessible quantitative metric based on the conventional sequence, rFLAIR was helpful to improve the survival prediction of posttreatment LGG patients. An early posttreatment MRI performed after treatment could be used for the delineation of tumor remnants in the region with non-enhancing high signal on FLAIR images. The combination of rFLAIR, clinical factors, and conventional MRI features may improve the survival prediction of LGG patients when a suspected non-enhancing high-signal lesion on FLAIR images is found.

## Data availability statement

The raw data supporting the conclusions of this article will be made available by the authors, without undue reservation.

## Ethics statement

The studies involving human participants were reviewed and approved by the ethical committee of The Second Hospital of Hebei University. The ethics committee waived the requirement of written informed consent for participation.

## Author contributions

TY, ZG, and GQ designed the study. TY, ZG,FW, J-LR, TW, HZ, GG, and GQ contributed equally to acquiring, analyzing, and interpreting the data and drafting the initial manuscript. ZG and TY performed the data analysis. TY, ZG and GQ made important revisions to the manuscript. TY and ZG contributed equally to this work. All authors contributed to the article and approved the submitted version.

## Funding

This study is supported by the Specialist Leadership Project of Hebei Province (No. 361004, GQ), Technology tracking program for medical application of Hebei Province (No. G201725, GQ)

## Conflict of interest

Author J-LR was employed by GE Healthcare.

The remaining authors declare that the research was conducted in the absence of any commercial or financial relationships that could be construed as a potential conflict of interest.

## Publisher’s note

All claims expressed in this article are solely those of the authors and do not necessarily represent those of their affiliated organizations, or those of the publisher, the editors and the reviewers. Any product that may be evaluated in this article, or claim that may be made by its manufacturer, is not guaranteed or endorsed by the publisher.
